# Unmasking three blinding indices for randomized controlled trials: comparison and application

**DOI:** 10.1016/j.conctc.2025.101553

**Published:** 2025-09-16

**Authors:** Javier Muñoz Laguna, Jafar Kolahi, Heejung Bang

**Affiliations:** a Musculoskeletal Epidemiology Research Group, Epidemiology, Biostatistics and Prevention Institute (EBPI) and University Spine Centre Zurich (UWZH), University of Zurich and Balgrist University Hospital, Zurich, Switzerland; b Epidemiology, Biostatistics and Prevention Institute (EBPI), University of Zurich, Zurich, Switzerland; c University Spine Centre Zurich (UWZH), Balgrist University Hospital, University of Zurich, Zurich, Switzerland; d Independent Research Scientist, Isfahan, Iran; e Division of Biostatistics, Department of Public Health Sciences, University of California, Davis, California, United States of America

**Keywords:** Blinding assessment, Clinical trial, Double-blind method, Masking, Single-blind method

## Abstract

Blinding is an important methodological aspect of randomized controlled trials. Although three blinding indices are available for the estimation of blinding in clinical trials, little guidance is available regarding their comparison and application. Here, we compared three blinding indices and applied them to hypothetical and real-world clinical trial data. Through this comparison and application tutorial, we identified strengths and limitations of each blinding index. Our findings provide insights into the assessment, estimation and reporting of blinding in randomized controlled trials.

## 1 Introduction

Blinding—or masking—is an important methodological aspect of randomized controlled trials (RCT) [1]. Blinding assessment may rely on asking different trial roles about their treatment beliefs after randomization. From these treatment belief data, the success of blinding may be estimated using blinding indices (BIs).

For simplicity, we consider a participant randomly assigned to an active treatment (‘A’) group. In a blinding assessment questionnaire, they may indicate they believed they received (i) an active treatment (‘I am in A’), (ii) a control (‘I am in B’), or (iii) report genuine uncertainty (‘I don't know’ [IDK]). These blinding data can be structured in the following 2x3 format, cross-tabulating treatment arm assignment against beliefs about treatment:

**Table:** 

**Arm**	**Beliefs**		
	**I am in A**	**I am in B**	**IDK**
A	n_AA_	n_BA_	n_DA_
B	n_AB_	n_BB_	n_DB_

where n_ij_ is the number of participants who indicated they believed they received treatment i and were randomly assigned to arm j (i,j = A or B), with i = D denoting IDK responses.

Although three BIs are available for the estimation of blinding in clinical trials, little guidance is available regarding their comparison and application. The James and Bang BIs have been endorsed by guidelines [[2], [3], [4], [5]]. They have also been used in multiple RCTs, systematic reviews, and meta analyses [[6], [7], [8]]. Recently, a Simple BI was proposed to improve existing methods [9].

In this tutorial, we aimed to (i) compare the formulae and characteristics of three BIs (James, Bang and Simple), and (ii) apply them to hypothetical and real-world clinical trial data.

## 2 Methods

### 2.1 Three BIs for RCTs

Below, we define and compare the formulae of three BIs—James (JBI), Bang (BBI) and Simple (SBI)—in chronological order. We use BI for parameter/estimand as well as estimator/estimate interchangeably for efficient communication. Variance, standard error, and/or confidence interval (CI) formulae can be found in the original publications [4,5,9].

### 2.2 James Blinding Index (JBI)

James BI was proposed in 1996 [4], and can be estimated by:

(1) JBI=[1+PˆD+(1–PˆD)∗{(PˆO–PˆE)/PˆE}]2;PˆO=∑i=AB∑j=ABwijPˆij(1–PˆD),andPˆE=∑i=AB∑j=ABwijPˆi.(Pˆ.j–PˆDj)(1–PˆD)2,whenPD≠1,

where parameter P_ij_ can be estimated by observed data n_ij_/N, P_Dj_ by n_Dj_/N, and P_D_ by (n_DA_ + n_DB_)/N.

Here, the total sample size is N = n_AA_ + n_BA_ + n_DA_ + n_AB_ + n_BB_ + n_DB_, and standard hat- and dot-notations denote estimator/estimate and summation over subscript, respectively. P_O_ and P_E_ represent weighted proportion of *observed* and *expected* guesses, respectively, and the term (P_O_–P_E_)/P_E_ is comparable to the kappa statistic. w_ij_ denotes constant weight for cell (i,j), taking value of 0 for correct, 0.5 for incorrect, and 1 for IDK. Accordingly, IDK responses have the greatest influence on the James BI. In an extreme case where P_D_ = 1 (all responses are IDK, no variability), JBI yields a value of 1 or undefined due to a ‘division by zero’ term.

### 2.3 Bang Blinding Index (BBI)

Bang BI was proposed in 2004 and can be written in three components as (i) BBI_A_ for arm A, (ii) BBI_B_ for arm B, and (iii) sumBI at the study level:

(2) $$\begin{array}{l} {BBI}_{A}={\hat{P}}_{A|A}-{\hat{P}}_{B|A}{=n}_{AA}/\left(n_{AA}{+n}_{BA}{+n}_{DA}\right)-n_{BA}/\left(n_{AA}{+n}_{BA}{+n}_{DA}\right)\text{,}\\ {BBI}_{B}={\hat{P}}_{B|B}-{\hat{P}}_{A|B}{=n}_{BB}/\left(n_{AB}{+n}_{BB}{+n}_{DB}\right)-n_{AB}/\left(n_{AB}{+n}_{BB}{+n}_{DB}\right)\text{,}\\ {\mathrm{SumBI}=\mathrm{BBI}}_{A}+{\mathrm{BBI}}_{B} \end{array}$$

where P_i|j_ is the probability of guessing treatment i when arm j is assigned [5].

BBI was originally considered for a simpler case without IDK responses:

$$\begin{array}{l} {BBI}_{A}{=2*n}_{AA}/\left(n_{AA}{+n}_{BA}\right)–1\text{,}\\ {BBI}_{B}{=2*n}_{BB}/\left(n_{AB}{+n}_{BB}\right)–1\text{,} \end{array}$$

and extended to cases where IDK responses are allowed:

(3) $$\begin{array}{l} {BBI}_{A}=\left[2*n_{AA}/\left(n_{AA}{+n}_{BA}\right)-1\right]*\left(n_{AA}{+n}_{BA}\right)/\left(n_{AA}{+n}_{BA}{+n}_{DA}\right)\text{,}\\ {BBI}_{B}=\left[2*n_{BB}/\left(n_{AB}{+n}_{BB}\right)-1\right]*\left(n_{AB}{+n}_{BB}\right)/\left(n_{AB}{+n}_{BB}{+n}_{DB}\right)\text{.} \end{array}$$

As such, equations (2) and (3) are equivalent except in an extreme case where all responses are IDK.

As opposed to JBI, BBI focuses on the balance of guesses within and between arms, instead of IDK responses.

### 2.4 Simple Blinding Index (SBI)

SBI was recently proposed in 2024 [9] as:

(4) $$\text{SBI}={\hat{P}}_{A}-{\hat{P}}_{B}=\frac{n_{AA}}{\left(n_{AA}+n_{BA}\right)}-\frac{n_{AB}}{\left(n_{AB}+n_{BB}\right)}$$

where P_A_ and P_B_ are the probability of guessing active treatment among participants in active and control arms, respectively. Importantly, SBI discourages the inclusion of IDK as a response option in blinding questionnaires.

### 2.5 Comparison of formulae and characteristics

Per the above formulae, a JBI of 1, a BBI or sumBI of 0, and an SBI of 0 indicate *perfect* blinding. However, there are nuances that should be noted when comparing the characteristics of the three BIs. First, JBI measures disagreement beyond chance, scaled between 0 and 1 [4]. Second, BBI and SBI are *contrasts* of binomial or multinomial probabilities with a null value of 0 for symmetry [5,9,10]. Of note, the similarities between SBI and SumBI are apparent from their formulae. Third, JBI and SBI are unique study-level indices of blinding, whereas BBI represents two arm-specific indices (BBI_A_ and BBI_B_), supplemented by one study-level index (sumBI).

Table 1 provides a summary of the characteristics of the three BIs.

**Table 1:** Characteristics of three blinding indices.

Characteristic	James Blinding Index	Bang Blinding Index	Simple Blinding Index
**Year**	1996	2004	2024

**Formulae** a	JBI = [1+P_D_+(1–P_D_)∗{(P_O_–P_E_)/P_E_}]/2, where P_O_ = $\sum_{i=A}^{B}\sum_{j=A}^{B}w_{ij}$ P_*ij*_/(1–P_D_), P_E_ = $\sum_{i=A}^{B}\sum_{j=A}^{B}w_{ij}$ P_*i*_*.*(P._*j*_–P_Dj_)/(1–P_D_)^2^	BBI_A_=P_A|A_–P_B|A_ BBI_B_=P_B|B_–P_A|B_ SumBI=BBI_A_ + BBI_B_	SBI=P_A_–P_B_

**What does it measure?**	Disagreement beyond chance across arms	Proportion of correct guesses beyond chance within an arm (BBI_A_ and BBI_B_) Between-arm difference in proportions of *same guess*^b^ (SumBI)	Between-arm difference in proportions of active treatment guess

**Trial design**	Two or more arms	Two arms and 1:1 allocation ratio	Two arms and any allocation ratio

**Blinding data format** c	2x2, 2x3, 2x5 for two arms Three or more arms allowed	2x2, 2x3, 2x5	2x2

**Range**	0 to +1	−1 to +1	−1 to +1

**Perfect value**	1	0	0

**Blinding cutoff (ad hoc)**	>0.5	<|0.2| (stringent) or <|0.3| (less stringent)	Not available yet

**Strengths**	One value Simple index scaled between 0 and 1 Can handle designs with more than two arms Intuitive if users are familiar with kappa statistic	Arm-specific and study-level indices Can capture *random guess* within an arm and *same guess* between arms Intuitive if users are familiar with multinomial distributions	One value Simplest, intuitive measure of *same guess* of *active* treatment Robust to any allocation ratio Intuitive if users are familiar with binomial distributions

**Limitations**	Arm-specific behaviors cannot be captured Subjective weights required *Same guess* patterns are not handled well Not oriented for 2x2 blinding data format	Three values may be confusing; particularly, sumBI measures a between-arm *difference* despite being a *sum* Subjective weights for 2x5 blinding data formats Suboptimal for designs beyond two arms or beyond 1:1 allocation ratio Vague interpretation of IDK responses and opposite guesses	Arm-specific behaviors cannot be captured Genuine uncertainty (IDK) is not captured in questionnaire; analytical sample size may decrease due to missing data Suboptimal for designs beyond two arms

**Additional considerations**	Like kappa statistic, underlying formula can be less intuitive Jackknife-variance formula provided	One- or two-sided CIs are available, with choice of alpha	Interpretation guidance is not yet available Wilson score method for CI

Abbreviations: BI, blinding index; CI, confidence interval; IDK, I don't know.

a Formulae of estimands; see main text for notation of parameters and estimators.

b *Same guess* refers to scenarios where participants in both arms guess the same (e.g., active) treatment.

c Blinding data takes 2x2 format when IDK responses are not available in blinding questionnaires. Blinding data takes 2x5 format when responses are Likert-type.

## 3 Application

We applied the three BIs to two sets of data (Table 2). The first set is comprised of hypothetical data from a recent publication [9]. This set is instructive for this tutorial and elucidates differences between the three BIs. The second set represents real-world RCTs with different blinding scenarios [[11], [12], [13], [14], [15], [16]]. RCTs in this set were purposively selected to seek variation in terms of year of publication, country, design, and blinding characteristics. For BI estimation, we used available R packages [17,18].

**Table 2:** Application of three blinding indices to hypothetical and real-world clinical trial data.

Arm: Count for A, B, IDK guess responses	Blinding Index (95 % CI)	Interpretation
**A—Hypothetical data** [9]		

**A: 0, 0, 100** **B: 0, 0, 100**	JBI = 1 (1, 1) or Undefined BBI_A_/BBI_B_=Undefined/Undefined or 0 (−1, 1)/0 (−1, 1) sumBI=Undefined or 0 (0, 0) SBI = 0 (0, 0)	*Perfect* blinding by JBI (if 1), BBI (if 0) and SBI (if 0). No value by BBI in R [17]. Undefined value (e.g., NaN in R) due to division by zero can happen for JBI and BBI depending on formula used.

**A: 0, 100, 0** **B: 100, 0, 0**	JBI = 1 (1, 1) BBI_A_/BBI_B_ = −1 (−1, −1)/-1 (−1, −1) sumBI = −2 (−2, −2) SBI = −1 (−1, −0.95)	*Perfect* blinding by JBI. *Opposite guess* in both arms by BBIs. Worst blinding by SBI.

**A: 50, 50, 0** **B: 50, 50, 0**	JBI = 0.5 (0.43, 0.57) BBI_A_/BBI_B_ = 0 (−0.20, 0.20)/0 (−0.20, 0.20) sumBI = 0 (−0.28, 0.28) SBI = 0 (−0.14, 0.14)	*Random guess* by JBI. *Perfect* blinding by BBI (or sumBI) and SBI.

**A: 50, 0, 50** **B: 0, 50, 50**	JBI = 0.5 (0.43, 0.57) BBI_A_/BBI_B_ = 0.5 (0.40, 0.60)/0.5 (0.40, 0.60) sumBI = 1 (0.86, 1.14) SBI = 1 (0.90, 1)	*Random guess* by JBI. Not ideal blinding by BBIs, sumBI and SBI.

**A: 15, 0, 85** **B: 0, 15, 85**	JBI = 0.85 (0.80, 0.90) BBI_A_/BBI_B_ = 0.15 (0.08, 0.22)/0.15 (0.08, 0.22) sumBI = 0.30 (0.19, 0.41) SBI = 1 (0.71, 1)	Successful blinding by JBI and BBI. Marginal unblinding may be judged by sumBI, and worst blinding by SBI.

**A: 36, 12, 19** **B: 18, 6, 9**	JBI = 0.64 (0.55, 0.73) BBI_A_/BBI_B_ = 0.36 (0.17, 0.54)/-0.36 (−0.63, −0.10) sumBI = 0 (−0.31, 0.31) SBI = −0 (−0.19, 0.22)	Successful blinding by JBI (lower limit of CI > 0.5). *Perfect* blinding by sumBI and SBI. CI width in BBI_B_ > BBI_A_ due to arm size.

**A: 50, 17, 0** **B: 25, 8, 0**	JBI = 0.51 (0.41, 0.60) BBI_A_/BBI_B_ = 0.49 (0.28, 0.70)/-0.52 (−0.81, −0.22) sumBI = −0.02 (−0.38, 0.34) SBI = −0.01 (−0.17, 0.18)	*Random guess* by JBI (point estimate ∼0.5); no IDK responses. Near *perfect* blinding by sumBI and SBI. CI width in BBI_B_ > BBI_A_ due to arm size.

**B—Real-world data**		

**Trial Characteristics: Treatment (A vs. B)/Primary outcome/Role assessed for blinding/Timing of assessment/Year/Country [Reference]**		

**Artificial intelligence vs. sonographers/initial interpretation/cardiologists/immediate/2023/USA** [11]		

**A: 557, 427, 756** **B: 418, 573, 764** a	JBI = 0.68 (0.66, 0.69) BBI_A_/BBI_B_ = 0.07 (0.04, 0.11)/0.09 (0.05, 0.12) sumBI = 0.16 (0.13, 0.19) SBI = 0.14 (0.10, 0.19)	*Random guess* by JBI and BBI. Interpretation guidance for SBI is pending (i.e., unclear if 0.14 is large enough to suggest unblinding). Because data collection allowed IDK response options, SBI loses analytical sample size here and elsewhere.

**New vs. old violins/tonal superiority/renowned soloists/immediate/2014/France** [12]		

**A: 15, 18, 0** **B: 13, 18, 5**	JBI = 0.52 (0.40, 0.64) BBI_A_/BBI_B_ = −0.09 (−0.43, 0.25)/0.14 (−0.16, 0.44) sumBI = 0.05 (−0.18, 0.28) SBI = 0.04 (−0.20, 0.26)	Successful blinding suggested by all BIs. *Random guess* within an arm or *same guess* between arms.

**Low vs. high dose statin vs. placebo/lipid levels/participants/end of study/1994/USA** [13]^b^		

**A: 38, 44, 21, 4, 170** **B: 11, 16, 21, 8, 83** c **(among IDK group when re-asked,** **A: 79, 86** d **B: 36, 45)**	JBI = 0.75 (0.71, 0.78) BBI_A_/BBI_B_ = 0.16 (0.11, 0.21)/0.01 (−0.06, 0.08) sumBI = 0.16 (0.07, 0.25) SBI = 0.28 (0.13, 0.43)	JBI suggests successful blinding (lower limit of CI > 0.5) due to high proportion of IDK responses. *Random guess* in placebo arm, but more correct guesses in statin arm by BBI; arm-specific blinding may be captured better by BBIs. SBI may not suggest successful blinding; interpretation guidance for SBI is pending.

**Spinal manual therapy vs. placebo therapy/blinding efficacy/outcome assessors/seven days after randomization/2025/Switzerland** [14]		

**A: 0, 4, 3, 0, 32** **B: 0, 6, 3, 0, 32**	JBI = 0.91 (0.85, 0.97) BBI_A_/BBI_B_ = 0.03 (−0.11, 0.16)/−0.07 (−0.21, 0.07) sumBI = −0.04 (−0.23, 0.15) SBI = −0.1 (−0.48, 0.32)	Successful blinding suggested by all BIs. *Random guess* within an arm or *same guess* between arms.

**Active vs. sham acupuncture/diabetic peripheral neuropathy/participants/immediate/2010/China** [15]		

**A: 42, 0** e **B: 19, 2**	JBI = 0.44 (0.36, 0.52) BBI_A_/BBI_B_ = 1 (1, 1)/−0.81 (−1.06, −0.56) sumBI = 0.19 (0.06, 0.32) SBI = 0.1 (−0.01, 0.29)	JBI suggests blinding may not be successful; JBI does not handle 2x2 blinding data format well. SumBI and SBI may suggest successful blinding; *same guess* pattern is better captured by these indices. Interpretation guidance for SBI is pending.

**Low vs. high dose disulfiram vs. riboflavin/alcoholism/coordinators/one year follow-up/1984/USA** [16]		

**A: 41, 66, 30, 44** f **B: 27, 72, 24, 51** **C: 22, 36, 64, 52** **(original data)** **A: 206, 54, 95** **B: 58, 64, 52** **(two arms combined)**	JBI = 0.56 (0.52, 0.59)^g^ When A and B arms are combined (2x3), JBI = 0.52 (0.48, 0.57) BBI_A_/BBI_B_ = 0.43 (0.35, 0.51)/0.03 (−0.09, 0.16) sumBI = 0.46 (0.31, 0.61) SBI = 0.32 (0.21, 0.41)	JBI suggests *random guess* (point and lower limit of CI ∼0.5); JBI is optimal for analyzing 3x4 blinding data format. BBI and SBI do not handle 3x4 blinding data format, so two arms were combined. BBI, sumBI and SBI suggest blinding may be somewhat not successful; BBI for the control arm indicates some success but BBI cannot handle 2:1 allocation ratio correctly.

Abbreviations: BBI, Bang blinding index; CI, confidence interval; IDK, I don't know; JBI, James blinding index; SBI, Simple blinding index.

For simplicity, ‘A’ represents active treatment and ‘B’ represents control treatment.

Six counts represent n_AA_, n_BA_, n_DA_ in the first row and n_AB_, n_BB_, n_DB_ in the second row unless otherwise specified.

With *perfect* blinding, JBI = 1; BBIs = 0 or sumBI = 0, and SBI = 0. For BBI, two values are for arm A/B.

For SBI estimation, ‘strong’ and ‘somewhat’ response options were combined, and IDK responses excluded as SBI uses 2x2 blinding data format.

Immediate means shortly after intervention session; Year is year of publication; CIs are two-sided with alpha 0.05.

a True data obtained from authors. We derived data A: 545, 415; 780; B: 430, 585, 760 from reported numbers in the publication. True and derived data yielded identical results in up to two decimal points. Frequently, authors do not report data that are sufficient to compute BIs and are unable to provide data upon request. Thus, assumptions need to be made to construct data in 2x2 or 2x3 format. This scenario emulates this situation.

b High and low dose groups were combined, resulting in ∼2:1 allocation ratio.

c Five counts are for ‘Strongly believe I received A’, ‘Somewhat believe I received A’, ‘Somewhat believe I received B’, ‘Strongly believe I received B’, and IDK, respectively.

d Two counts of ‘I am in A' and ‘I am in B’ since IDK group was re-asked to guess a treatment. Participants who originally selected IDK but declined to respond when re-asked were excluded.

e Data collected in 2x2 format.

f Three rows are for low-dose disulfiram (1 mg), high-dose disulfiram (250 mg), and riboflavin arms, respectively. In each row, four values are for guesses of low-dose disulfiram, high-dose disulfiram, riboflavin, and IDK, respectively.

g Jacknife or asymptotic variance estimate yielded identical results.

To complement the first set, we assessed BI trends under additional hypothetical blinding scenarios (sFig. 1, Appendix A).

### 3.1 Hypothetical data

Table 2A shows seven hypothetical blinding scenarios illustrating differences in the three BIs. It is apparent that successful blinding based on JBI is highly influenced by the proportion of IDK responses. SumBI and SBI yield similar estimates due to comparable *between-arm* comparisons.

### 3.2 Real-world RCT data

Table 2B shows the application of the three BIs to blinding data from six real-world RCTs.

Here, we use the acupuncture RCT as an illustrative, noteworthy example [15]. In this trial, 42 (100 %) participants in the active arm correctly guessed that they received active acupuncture. Among participants randomly allocated to the control arm, 19 (90 %) guessed that they received active acupuncture. JBI suggests blinding may not be successful, partly due to the lack of IDK response option in the blinding questionnaire. BBI for Arm A yields a value of 1, suggesting complete unblinding. Yet, sumBI and SBI correct this naive interpretation at the study level, highlighting that even complete correct guesses within an arm may not be detrimental if there are comparable *same guess* proportions in *both* arms.

Of note, a zero-width CI for BBI is observed due to normality-based asymptotic CI [5]. This can also occur with JBI, although alternative variance/CI formulas (e.g., resampling-based) can address this problem [4,11]. By employing the Wilson score method, SBI avoids symmetry constraints and helps prevent zero-width CIs [9,18].

### 3.3 Strengths and limitations of three BIs for RCTs

We identified strengths of each BI. JBI can handle more than two arms with more than three guess response options, offering flexibility in application. BBI can capture arm- and study-level blinding scenarios. SBI is intuitive and can handle different allocation ratios.

We also identified weaknesses. JBI does not capture arm-specific blinding scenarios and does not handle *same guess* patterns between arms or 2x2 blinding data format well. BBI takes three complementary values, which may create challenges in interpretation and communication. BBI is also suboptimal for complex RCT designs beyond two arms and 1:1 allocation ratio. SBI does not capture arm-specific blinding scenarios and is suboptimal for RCTs with more than two arms. In addition, SBI does not collect genuine uncertainty in blinding questionnaires as IDK responses are omitted, which may generate missing data (see Refs. [[11], [12], [13], [14], [15], [16]], Table 2B).

## 4 Concluding remarks

In this tutorial, we compared three BIs and highlighted how differences among these are explainable by underpinning formulae and characteristics. Through application to hypothetical and real-world RCT data, we identified strengths and limitations of each BI. Our findings provide insights into the assessment, estimation and reporting of blinding in RCTs.

Regardless of the BI of choice, challenges remain on the estimation of bias attributable to beliefs about treatment within a specific trial. Yet, in RCTs where beliefs about treatment are *independent* of arm assignment, bias in effect estimates is often negligible *numerically* [19,20].

Our work illustrates that the three BIs can be complementary. Since blinding success is not binary, we underscore that point estimates and CIs are more informative than statistical tests, p-values, or overreliance on blinding cutoffs. For blinding related procedures, we recommend that trialists use the most appropriate methods, in conjunction with relevant reporting guidelines [2,3]. When feasible, and to further foster methodological rigor, trialists may prespecify their choice of primary and secondary BI in their trial protocol.

Our work has limitations. Our BI comparison was qualitative and did not include a comprehensive evaluation of numerical properties. In addition, our selection of hypothetical and real-world RCT data was ad hoc. Future research may consider building on this work by using simulation studies and applying the three BIs to broader RCT sets.

Trialists should work together with patient partners and other relevant stakeholders on the co-design of high-quality RCTs that consider blinding assessment, estimation and transparent reporting. A more thoughtful consideration of blinding is warranted to enhance the quality and integrity of RCTs, ultimately improving trust between researchers and the general public.

## CRediT authorship contribution statement

**Javier Muñoz Laguna:** Writing – review & editing, Investigation, Formal analysis. **Jafar Kolahi:** Writing – review & editing, Investigation, Formal analysis. **Heejung Bang:** Writing – original draft, Supervision, Methodology, Conceptualization.

## Funding

JML reports partial support from the Spanish Chiropractic Association (AEQ), the European Cooperation in Science and Technology (COST), and the Graduate Campus of the University of Zurich outside the submitted work. HB was partly supported by the National Institutes of Health through grants UL1 TR001860 and R61 AT012187. Funding sources had no role in the study design, implementation or submission of this work.

## Declaration of competing interest

The authors declare that they have no known competing financial interests or personal relationships that could have appeared to influence the work reported in this paper.

## Data Availability

Data will be made available upon reasonable request.
