# Supplementary material for: Unmasking three blinding indices for randomized controlled trials: comparison and application
Source: Contemp Clin Trials Commun. 2025 Sep 16;48:101553. doi: 10.1016/j.conctc.2025.101553 (PMC12547027; doi:10.1016/j.conctc.2025.101553)
Supplement: Multimedia component 1 [file mmc1.pdf]

Appendix A. Supplemental material.

sFigure 1. BI trends under additional hypothetical blinding scenarios.

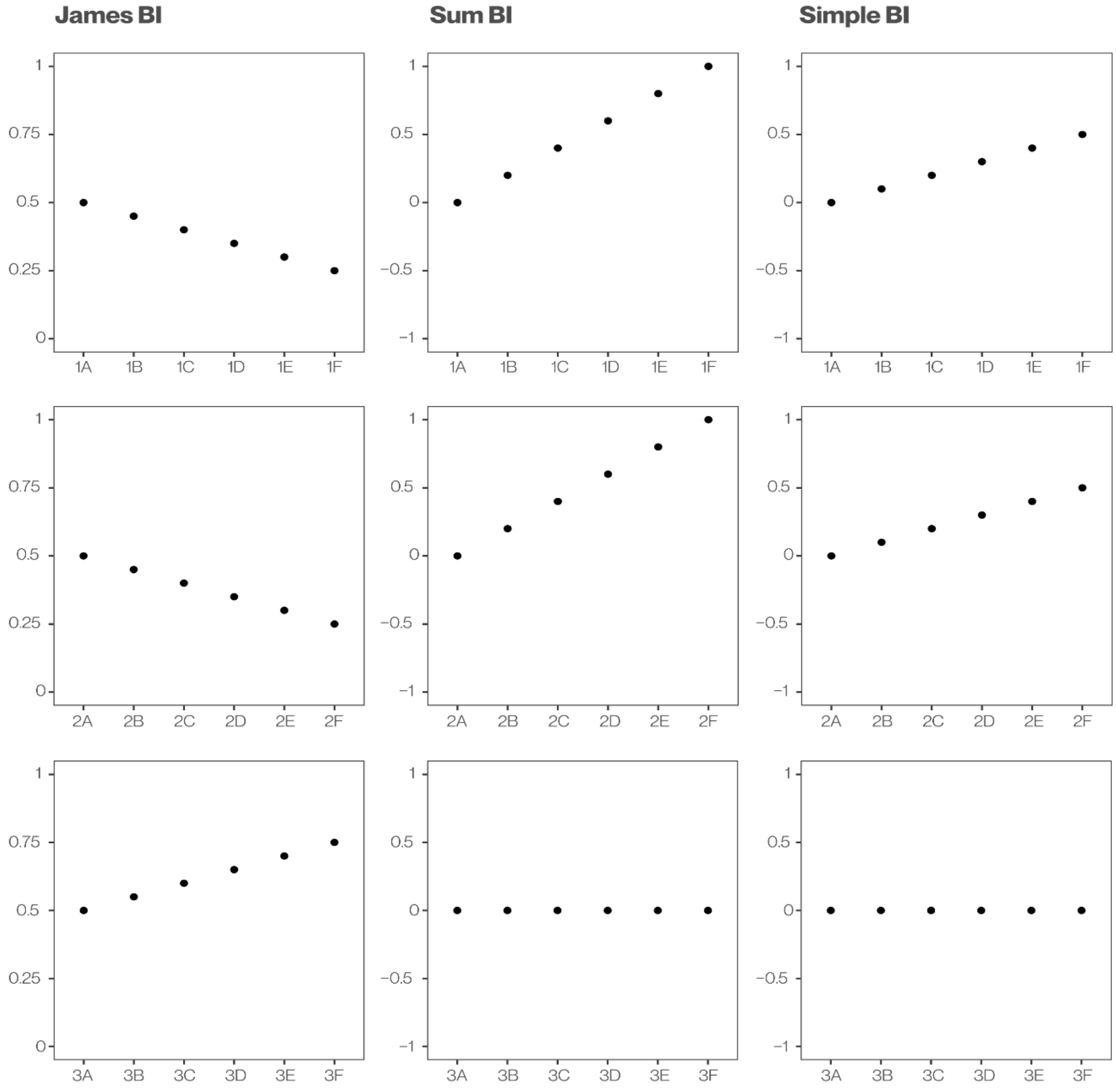

Scenarios 1A to **1F** (row 1) explore trends of three BIs under hypothetical blinding scenarios with increasing correct guesses in active arm, while holding guesses in control arm and IDK responses constant. **Scenarios 2A** to **2F** (row 2) explore trends of three BIs under hypothetical blinding scenarios with increasing correct guesses in control arm, while holding guesses in active arm and IDK responses constant. It should be noted that **Scenarios 1A** to **1F** and **2A** to **2F** are identical due to symmetry. **Scenarios 3A** to **3F** (row 3) explore trends of three BIs under hypothetical blinding scenarios with increasing IDK responses, while holding guesses in active arm and arms constant. Hypothetical data, N = 200.

Scenario 1A

Arm	Beliefs		
	A	B	IDK
A	50	50	0
B	50	50	0

Scenario 1B

Arm	Beliefs		
	A	B	IDK
A	60	40	0
B	50	50	0

Scenario 1C

Arm	Beliefs		
	A	B	IDK
A	70	30	0
B	50	50	0

Scenario 1D

Arm	Beliefs		
	A	B	IDK
A	80	20	0
B	50	50	0

Scenario 1E

Arm	Beliefs		
	A	B	IDK
A	90	10	0
B	50	50	0

Scenario 1F

Arm	Beliefs		
	A	B	IDK
A	100	0	0
B	50	50	0

Scenario 2A

Arm	Beliefs		
	A	B	IDK
A	50	50	0
B	50	50	0

Scenario 2B

Arm	Beliefs		
	A	B	IDK
A	50	50	0
B	40	60	0

Scenario 2C

Arm	Beliefs		
	A	B	IDK
A	50	50	0
B	30	70	0

Scenario 2D

Arm	Beliefs		
	A	B	IDK
A	50	50	0
B	20	80	0

Scenario 2E

Arm	Beliefs		
	A	B	IDK
A	50	50	0
B	10	90	0

Scenario 2F

Arm	Beliefs		
	A	B	IDK
A	50	50	0
B	0	100	0

Scenario 3A

Arm	Beliefs		
	A	B	IDK
A	50	50	0
B	50	50	0

Scenario 3B

Arm	Beliefs		
	A	B	IDK
A	45	45	10
B	45	45	10

Scenario 3C

Arm	Beliefs		
	A	B	IDK
A	40	40	20
B	40	40	20

Scenario 3D

Arm	Beliefs		
	A	B	IDK
A	35	35	30
B	35	35	30

Scenario 3E

Arm	Beliefs		
	A	B	IDK
A	30	30	40
B	30	30	40

Scenario 3F

Arm	Beliefs		
	A	B	IDK
A	25	25	50
B	25	25	50
